# Editorial: Epithelial-Mesenchymal Transition: Yet Another Exciting Avenue in Cancer Metabolic Remodeling

**DOI:** 10.3389/fonc.2020.628664

**Published:** 2021-01-28

**Authors:** Katarína Smolková, Péter Bai

**Affiliations:** ^1^Laboratory of Mitochondrial Physiology, Institute of Physiology of the Czech Academy of Sciences, Prague, Czechia; ^2^Department of Medical Chemistry, Faculty of Medicine, University of Debrecen, Debrecen, Hungary; ^3^MTA-DE Lendület Laboratory of Cellular Metabolism, Debrecen, Hungary; ^4^Research Center for Molecular Medicine, Faculty of Medicine, University of Debrecen, Debrecen, Hungary

**Keywords:** epithelial-mesenchymal transition, partial epithelial-mesenchymal transition, cancer mesenchymal phenotype, metabolic remodeling, cancer metabolism

Metastasis is a multistep process during which secondary tumors are formed in distant organs. The first step of a metastatic cascade is the epithelial-mesenchymal transition (EMT). EMT requires complex expression changes leading to the transformation of epithelial cells into mesenchymal ones, able to migrate. The changes in gene expression are brought about by a battery of well-characterized EMT-related transcription factors (EMT-TFs), responsible for the exchange of E-type cadherin into N-type cadherin, with a clearly defined role in promoting metastasis. The Research Topic “Role of Metabolic Remodeling in Cancer-Associated Epithelial-Mesenchymal Transition” aimed to gather information on metabolic features accompanying EMT, because conclusive information about cancer metabolic status in EMT is still rather elusive.

In general, metabolic remodeling is a “support tool” for the survival of cancer cells and successful progression. Our extensive knowledge and methodological advances in the field of cancer metabolism are now being continuously applied to discover specificities of other dynamic cancer-related processes, such as EMT. Apparently, an *in vitro* link between metabolic and mesenchymal phenotype is reciprocal; specific mesenchymal markers might be associated with the specific expression pattern of metabolic genes, and *vice-versa*, changes to the expression of some metabolic enzymes induce or inhibit EMT, respectively. Metabolic processes supporting EMT are similar to the general principles in cancer metabolism studied so far, *i.e.* metabolic signaling towards epigenetics, regulating cofactor abundance, or supporting metabolic plasticity of cancer cells.

Our article collection intended to point out relevant molecular links between the EMT process and specific metabolic enzymes or pathways. As stated above there are some links between a genetic and metabolic fragment of EMT influencing each other. A review by Georgakopoulos-Soares et al. wraps up information regarding the role of EMT-TFs in EMT and metabolic remodeling, and provides examples of how EMT-TFs possibly regulate metabolism. The authors summarize the consequences of EMT-TFs activity (Snail, Twist, and ZEB family) in EMT as well as in metabolism. The authors also focus on central pathways of carbon metabolism and the role of the individual enzymes in promoting or suppressing EMT in cancer. Metabolic changes can be also essential in drug resistance as shown in the article by Huang et al. High-glucose level inhibits the effect of 5-fluorouracil in colorectal carcinoma as elevated pyruvate levels in cytosol prevent necroptosis and scavenge excessive ROS.

The article collection also discusses the complexity of EMT stimuli in the tumor microenvironment and the complexity of cellular interactions, including cellular and non-cellular signaling. EMT-TFs and EMT are triggered in response to a variety of stimuli either intrinsic (signaling pathways, metabolic signaling), and extrinsic (hypoxia, pro-inflammatory signals, circulating metabolites), which is very adequately summarized in the review article of D´Angelo et al. The review also summarizes plethora of signals activating EMT by means of inter-cell communication within the stromal environment. Moreover, it brings up the innovative issue of extracellular vesicle production by mesenchymal cells and gathers evidence regarding the role of exosomes in metastasis initiation. The role of the tumor environment in populating a metastatic site, namely interaction of immune component, is highlighted in a mini-review by Fedele and Melisi, who describe strategies by which the immune compartment of cancer promotes EMT *via* inflammatory cytokines. Interestingly, as discussed, mesenchymal-type tumors are associated with immunosuppressive cytokines signature, which is a strategy that helps to overcome immunosurveillance.

However, an obvious drawback to study molecular mechanisms of EMT lies in the inability to entrap the population of cells undergoing EMT *in situ*, so that available studies are mostly based on the correlations and *in vitro* research. Hence, studies of metabolic arrangements at the single-cell level are limited to genetic and morphological methods. Our limitations to reach a clear conclusion toward pathology in cancer also lie in the existence of epithelial-mesenchymal plasticity, a so-called hybrid EMT state, implying that the EMT process is very elusive and difficult to capture in the experimental conditions, and might be tissue and context-dependent. The concept of a hybrid state of EMT, or partial EMT, is highlighted more or less intensively in the majority of the collected articles. A review article of Sun and Yang, again, summarizes the typical metabolic network affected in cancer and its potential use as a diagnostic and therapeutic marker, and points out several recent lines of evidence correlating EMT plasticity to cancer metabolism. The mesenchymal state is also perceived as a factor promoting chemotherapy resistance as well as a footstep to pluripotent mode. The summary of extrinsic and intrinsic signals leading to transcriptional regulation of EMT in terms of partial EMT phenotype and epigenetic signaling is provided in a review article by Lavin and Tiwari, which points out that patterns of epigenetic changes related to EMT might differ according to external stimuli, including hypoxia or TGF-β signaling. The authors are highlighting common ground, convergent signals in epigenetics heterogeneity during EMT and partial EMT, and between distinct EMT types. Another very innovative model of EMT and MET regulation is described in the mini-review by Stone, who draws attention to the intriguing concept of dependence and guidance receptors (DGRs). The receptors are able to induce anoikis as a physiological protective process in detached cells. Apparently, DGRs not only induce EMT but, netrin-DGR interactions regulate cell migration and the re-population of the distant tissues based on local netrin concentration. The article discusses the importance of observing the expression of particular EMT markers, which characterize partial EMT (mixed EMT phenotype).

To conclude, existing systematic research on the intersection of metabolic remodeling and EMT mostly surrounds the central carbon metabolism, as summarized in the collected articles. The originally dichotomic process of EMT, a transitioning from an epithelial to a mesenchymal phenotype, has become more complex by the discovery of partial EMT, or the discovery of its reversal (mesenchymal-to-epithelial transition, MET). The complexity is further aggravated by accumulating evidence pointing out to numerous proteins outside the central carbon metabolism clearly promoting cell mobility, such as BCAT1 (1) or LACTB (2), regulatory pathways linking metabolism to EMT, such as PGC-1 (3) or NRF2 (4), and even extrinsic factors, such as oncobiotic cues (5, 6). Our current understanding of these pathways is far from complete and warrants further studies explaining how metabolism contributes to cancer pathology *via* the metastasis initiation process. As a future prospect, it is necessary to address several fundamental questions; *i.e.* the matter of specific metabolic requirements of cancer cells to initiate EMT, or, *vice versa*, if specific metabolic “checkpoints” commit cells to EMT. Yet again, molecular mechanisms need to be elucidated to understand how to translate EMT-related metabolic targets into diagnostic and prognostic tools, or even into therapeutic targets using the principles of synthetic-lethal vulnerability.

## Author Contributions

KS conceptualized and reviewed the text along with PB. Both authors contributed to the article and approved the submitted version.

## Funding

KS was supported by grant 20-00408S of the Czech Science Foundation, NV19-01-00101 of the Czech Health Research Council, and by institutional support RVO:67985823. PB was supported by TKP2020-IKA-04, K123975 and GINOP-2.3.2-15-2016-00006 from the NKFIH.

## Conflict of Interest

The authors declare that the research was conducted in the absence of any commercial or financial relationships that could be construed as a potential conflict of interest.
